# Supplementary material for: Compact artificial neuron based on anti-ferroelectric transistor
Source: Nat Commun. 2022 Nov 17;13:7018. doi: 10.1038/s41467-022-34774-9 (PMC9668812; doi:10.1038/s41467-022-34774-9)
Supplement: Supplementary file 1 — Supplementary Information [file 41467_2022_34774_MOESM1_ESM.pdf]

Supplementary Information

Compact artificial neuron based on anti-ferroelectric transistor

Cao et al.

Supplementary Figures 1−11.

Supplementary Figures

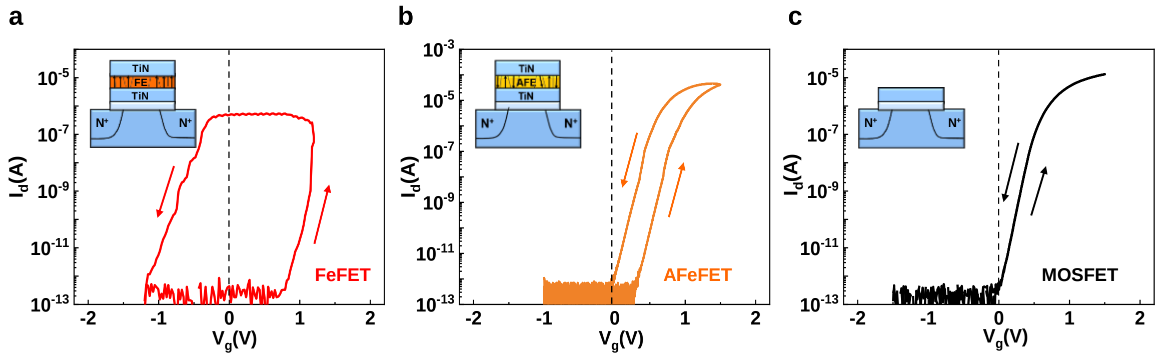

Supplementary Figure 1. Volatility of AFeFET. a Typical transfer curves of FeFET. **b** Typical transfer curves of AFeFET. **c** Typical transfer curves of MOSFET. The sweeping DC voltage is applied to the gate of FeFET, and 0.3 V DC voltage is applied to the drain terminal during measurement. The arrows indicate the switching sequences. Compared to MOSFET, both the transfer curves of FeFET and AFeFET exhibit counterclockwise hysteresis due to the polarization switching of $Hf_xZr_{1-x}O_2$ films under the electric field. But the transfer curve of AFeFET differs from that of FeFET at V_g=0 V, indicating that the AFeFET is volatile. Moreover, the AFeFET is electroforming-free.

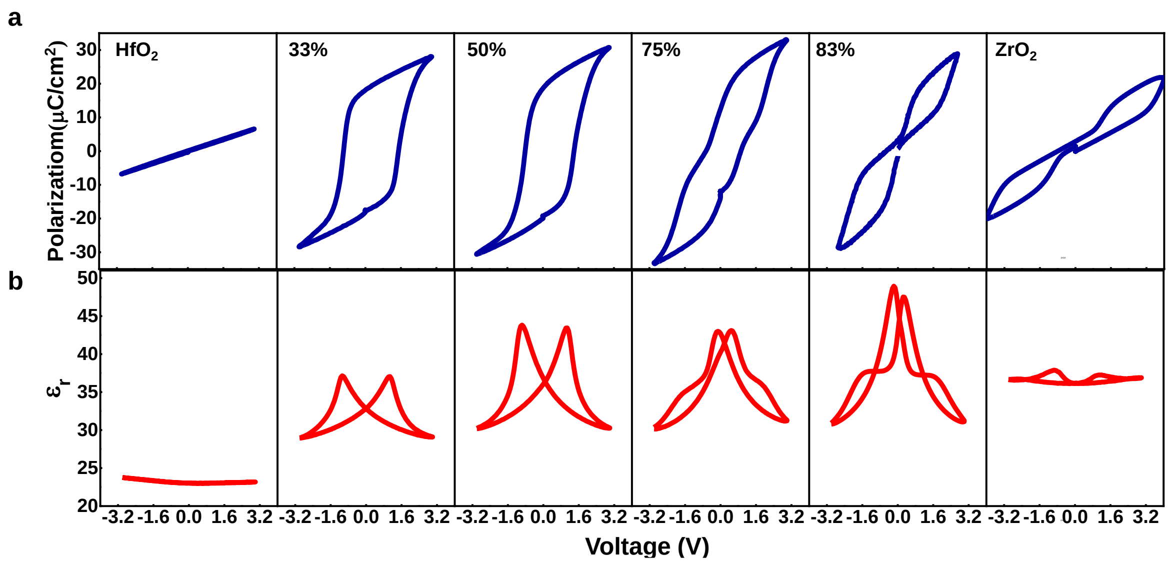

Supplementary Figure 2. The composition-dependent polarization-voltage (PV) and dielectric constant-voltage (CV) curves of the $Hf_xZr_{1-x}O_2$ MIM structures with TiN as top and bottom electrodes. a PV curves of $Hf_xZr_{1-x}O_2$ MIM structures with different zirconium concentrations as 0% (pure HfO_2), 33%, 50%, 75%, 83%, 100% (pure ZrO_2), indicating the paraelectric-ferroelectric-antiferroelectric evolution process of $Hf_xZr_{1-x}O_2$ films with the increasing zirconium content. The paraelectric HfO_2 exhibits a linear electric polarization dependence on the applied voltage. The ferroelectric (zirconium concentration as 33%, 50%) and antiferroelectric (zirconium concentration as 75%, 83%, 100%) $Hf_xZr_{1-x}O_2$ exhibit nonlinear electric polarization dependence on the applied voltage. The polarization loop of ferroelectric is a hysteresis loop, while the antiferroelectric MIM hows double hysteresis loop. **b** CV curves of $Hf_xZr_{1-x}O_2$ MIM structures with different zirconium concentrations (0%, 33%, 50%, 75%, 83%, 100%). The paraelectric HfO_2 exhibits a 0voltage-independent dielectric constant value. The CV of ferroelectric $Hf_xZr_{1-x}O_2$ exhibits a butterfly shape, while the antiferroelectric $Hf_xZr_{1-x}O_2$ exhibits a double butterfly shape.

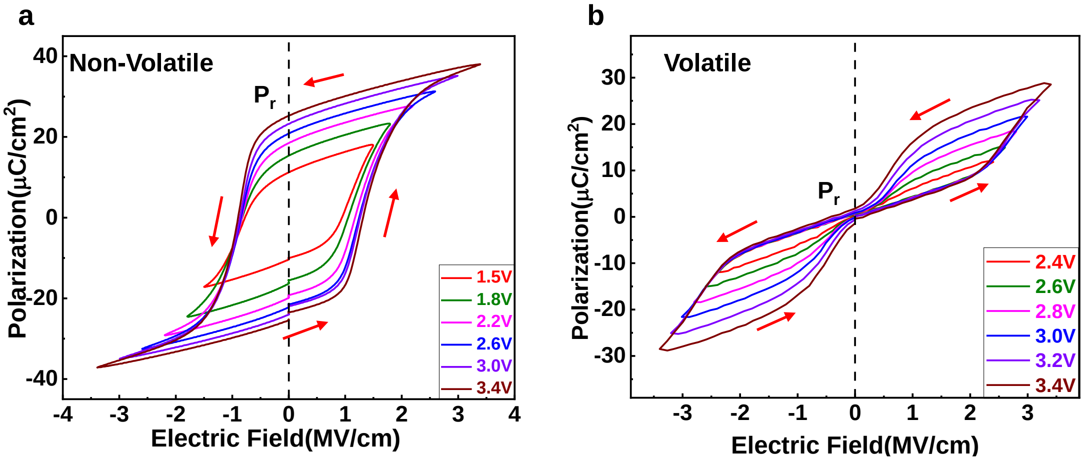

Supplementary Figure 3. Polarization-voltage curves of FE ($Hf_{0.5}Zr_{0.5}O_2$) and AFE ($Hf_{0.2}Zr_{0.8}O_2$) MIM structures under different voltages. a The FE MIM structure shows non-volatile PV curves. **b** the AFE MIM structure shows volatile PV curves. The polarization of AFE increases under a higher electric field, but it still can revert to its initial state when the applied electric field is removed. The arrows indicate the switching sequences.

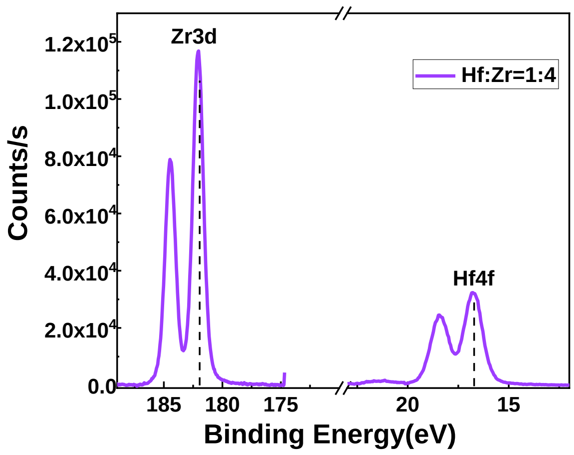

Supplementary Figure 4. The XPS results of $Hf_{0.2}Zr_{0.8}O_2$. The AFE $Hf_xZr_{1-x}O_2$ film composition is confirmed as hafnium: zirconium≈ 1: 4.

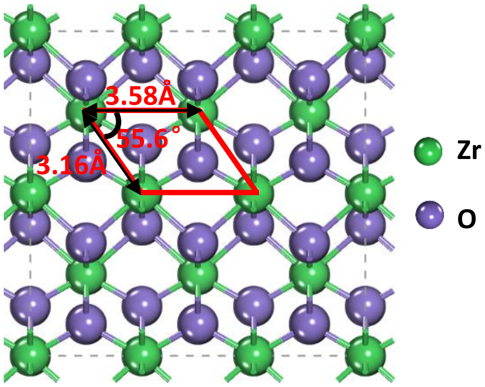

Supplementary Figure 5. The atomic model of [0-10] oriented *P4₂/nmc* structure. The relative angle and lattice constants match the experimental atomic structure in Fig. 2f.

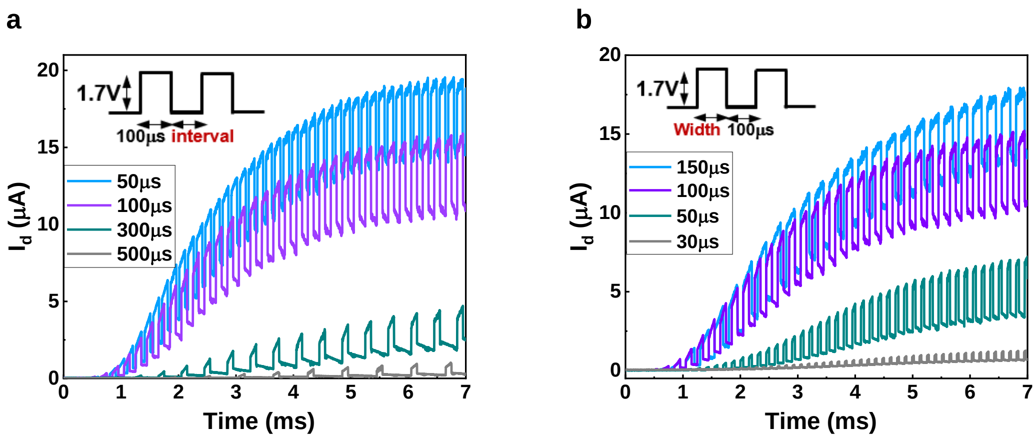

Supplementary Figure 6. The tendencies of I_d under continuous gate pulses with different gate pulse parameters. a Different gate pulse intervals (fixed 1.7 V amplitude, 50 μs-500 μs interval, fixed 100 μs width). The I_d increases faster and a larger saturation value in the case of shorter pulse intervals, as shorter intervals will limit the recover duration of AFE domains and contribute to the I_d increase. **b** Different gate pulse widths (fixed 1.7 V amplitude, fixed 100 μs interval, 30 μs-150 μs width). The larger pulse width corresponds to a larger I_d saturation value due to more AFE *t*-phase domains can be switched to FE domains under longer integration time.

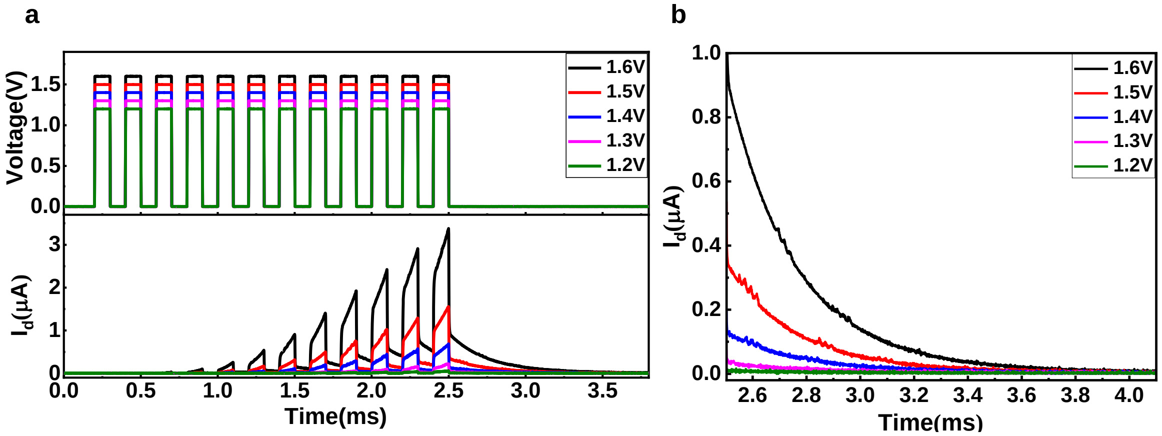

Supplementary Figure 7. The leakage process of AFeFET neuron with different I_d. a The LIF process of AFeFET neuron under 12 gate pulses with different amplitudes(1.2 V-1.6 V). The larger gate pulse amplitude results in higher I_d, and longer recovery time. **b** A closer view of leakage process with different I_d. The higher I_d of the AFeFET neuron needs a longer time to leak completely. On the whole, the recovery time of the AFeFET neuron is in the order of milliseconds.

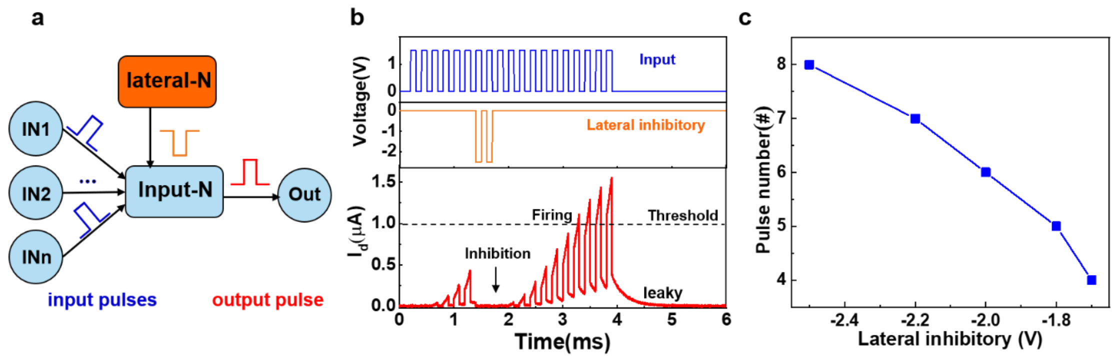

Supplementary Figure 8. The lateral inhibitory property of AFeFET neuron. a Schematic illustration of the lateral inhibitory. Negative gate pulses as inhibitory stimuli from adjacent neurons are applied during a series of excitatory input spikes (100 μs width, 100 μs interval, 1.5 V amplitude). **b** The response of AFeFET neuron to inhibitory stimuli. The excitability is inhibited once receiving inhibitory stimuli (100 μs width, 100 μs interval, -2.5 V amplitude) from adjacent neurons. After inhibition, the AFeFET neuron gets excitive again and fires under the continuous excitatory stimuli. **c** The pulse number for the next firing after receiving different inhibitory stimuli (-1.7 V，-1.8 V，-2 V，-2.2 V，-2.5 V). The AFeFET neuron needs more excitatory stimuli for the next firing with stronger inhibitive intensity.

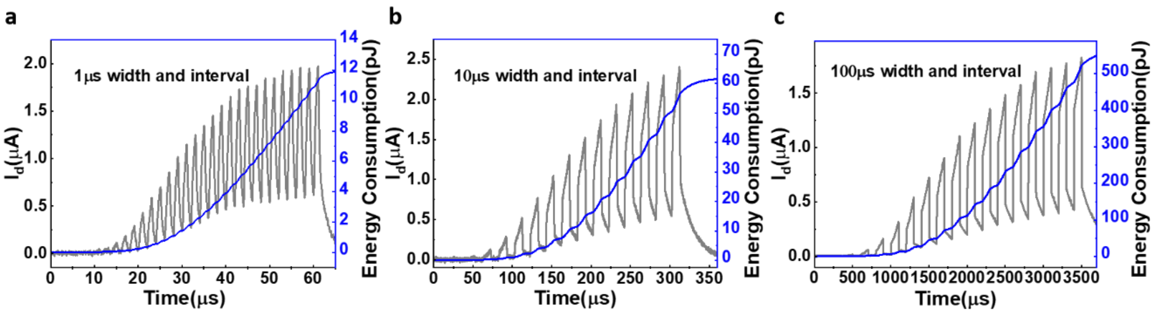

Supplementary Figure 9. The energy consumption per spike dependence on pulse parameters and the threshold value of AFeFET neurons. The energy consumption of AFeFET neurons with **a** 1 μs **b** 10 μs **c** 100 μs gate pulse width and interval. The energy consumption E_{AFeFET} is achieved by accumulating $I_d V_{ds} \Delta t$, the V_{ds} is 0.3V duing measurement. With the reduction of pulse width or threshold value, the energy consumption decreases and the measured lowest energy consumption is 37 fJ per spike (1μs width and interval, 50nA threshold). The energy consumption can be further reduced as pulse width and threshold decreasing.

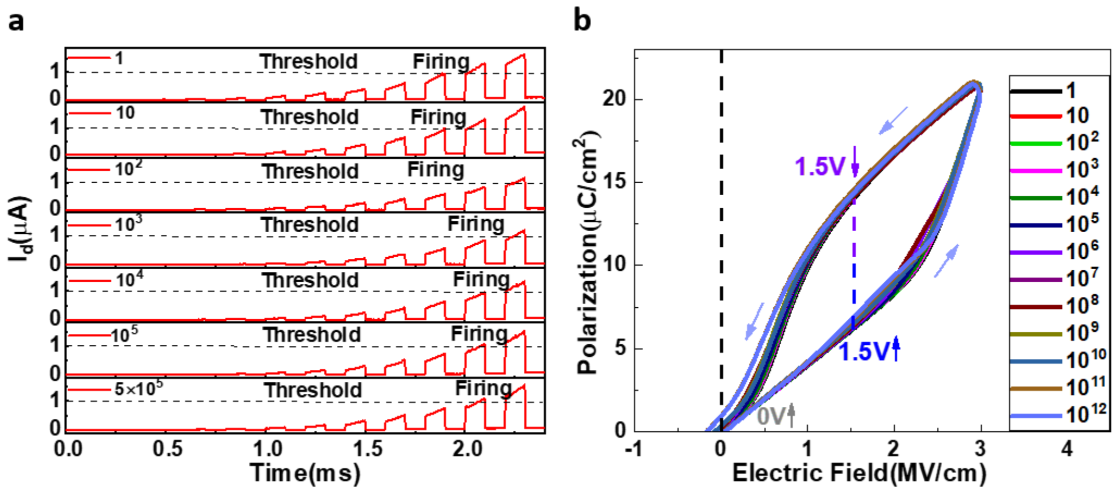

Supplementary Figure 10. The endurance measurements of AFeFET neurons. a The AFeFET neuron can fire 5×10^5 cycles stably without any significant deterioration. **b** In order to speed up the measurement, the AFE MIM structure, which is the endurance bottleneck of AFeFET, is measured for higher endurance (>10^{12} cycles).

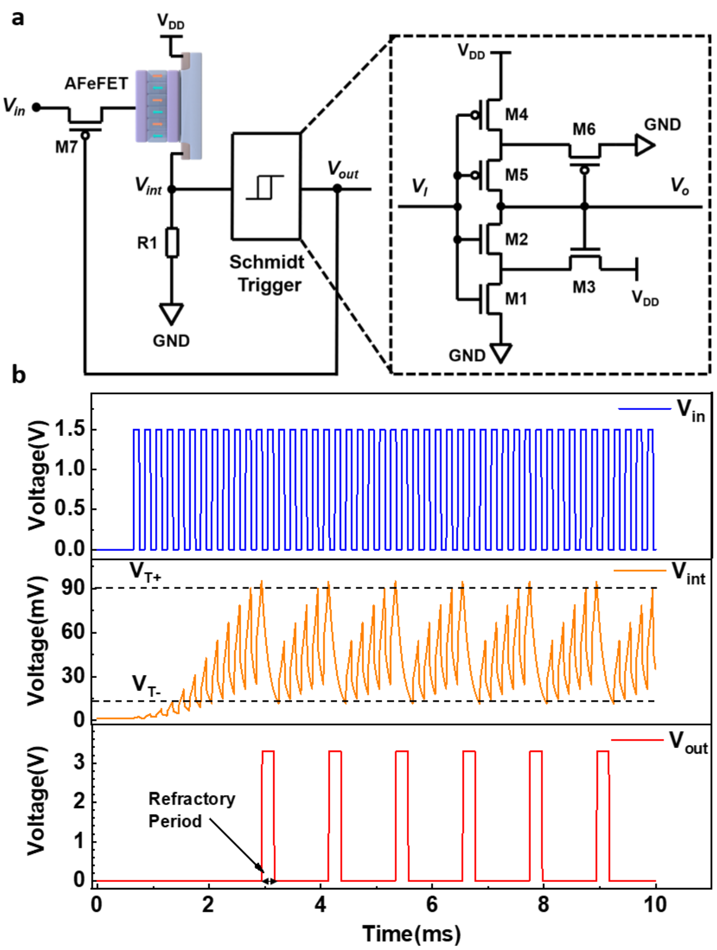

Supplementary Figure 11. a The circuit realization and **b** simulation results of a AFeFET neuron with refractory period and driven capability.

About the generation of spike at the output. The AFeFET is used to achieve the potential integration function, and the spike generation module is realized by using Schmidt trigger. The threshold voltages (V_{T-} aμnd V_{T+}) of Schmidt trigger are defined by the working parameters of M1, M2, M4 and M5. The R1 resistor is used to convert the current signal of AFeFET into voltage. Once the voltage of V_{int} is higher than V_{T+}, the output potential of Schmidt trigger will be pull up to V_{DD}, as shown in Supplementary Figure 11. b. When the voltage of V_{int} is lower than V_{T-}, the output potential of Schmidt trigger will be pull down to ground.

About the controllable refractory period. Once the voltage of V_{int} is higher than V_{T+}, the output potential of Schmidt trigger will be pull up to V_{DD}. The channel of M7 transistor

switches off when it received the output feedback signal and the input node V_{in} of AFeFET neurons is off, as shown in Supplementary Figure 11. b. Thus, the AFeFET neuron implements the controllable refractory period. The time of refractory period is controlled by the V_{T-} of Schmidt trigger and the discharge process of V_{int}.
